# New Paradigms of Extended Thromboprophylaxis in Medically Ill Patients

**DOI:** 10.3390/jcm9041002

**Published:** 2020-04-02

**Authors:** Kira MacDougall, Alex C Spyropoulos

**Affiliations:** 1 Department of Internal Medicine, Northwell Health at Staten Island University Hospital, Staten Island, NY 10305, USA; kmacdougall1@northwell.edu; 2 Center for Health Innovations and Outcomes Research, The Feinstein Institutes for Medical Research and Donald and Barbara Zucker School of Medicine at Hofstra/Northwell, Manhasset, NY 11030, USA; 3 Department of Medicine, Anticoagulation and Clinical Thrombosis Services, Northwell Health at Lenox Hill Hospital, 130 E 77th St., New York, NY 10075, USA

**Keywords:** venous thromboembolism, medically ill patients, direct oral anticoagulants, extended thromboprophylaxis

## Abstract

Extended thromboprophylaxis given to medically ill patients for up to 45 days following an acute hospitalization remains an emerging topic among many hospital-based health care providers. Recent advancements in the field of extended thromboprophylaxis using risk stratification and careful patient selection criteria have led to an improved safety profile of direct oral anticoagulants (DOACs) and established net clinical benefit when given to key patient subgroups at high risk of venous thromboembolism (VTE) and low risk of bleeding. The Food and Drug Administration (FDA) has now approved the DOACs betrixaban and rivaroxaban for both in-hospital and extended thromboprophylaxis in medically ill patients in these key subgroups, which represents more than one-quarter of hospitalized medically ill patients. This has potential to significantly reduce VTE-related morbidity and mortality for these patients. Emerging data also supports reductions in the risk of arterial thromboembolism in medically ill patients with extended thromboprophylaxis post-hospital discharge using DOACs. This article aims to review the most recent concepts of predicting and preventing VTE and to discuss emerging paradigms of extended thromboprophylaxis in hospitalized medically ill patients utilizing an individualized, risk-adapted approach.

## 1. Introduction

A significant proportion of the estimated 20 million hospitalized, acutely-ill medically-ill patients in the USA and EU are at risk of developing venous thromboembolism (VTE) [1,2]. Prophylactic treatments with unfractionated heparin (UFH), low-molecular-weight heparin (LMWH), and the pentassacharide fondaparinux have shown a 50%–60% reduction in total VTE given for 6–14 days [3,4,5]. However, hospital length-of-stay (LOS) in the USA now averages approximately 4.5 days and is decreasing in Canada and the European Union [6]. Despite consistent data that show more than 60% of all VTEs occur after the post-hospital discharge period and that the majority of VTE events (approximately 80%) occur within 6 weeks after discharge, post-hospital thromboprophylaxis is given to less than 4% of hospitalized medically-ill patients [7,8].

Until recently, the topic of extended thromboprophylaxis with direct oral anticoagulants (DOACs) for up to 45 days in hospitalized medically ill patients has been a controversial one. At least 25% of all medically ill patients are at sufficient VTE risk to benefit from extended thromboprophylaxis, but earlier placebo-controlled randomized trials with enoxaparin, and the DOACs rivaroxaban and apixaban, failed to establish a definitive net clinical benefit in overall populations due to an increase in bleeding [9,10,11]. Data from meta-analyses supported an approximate 40% decrease in symptomatic VTE and VTE-related death with extended thromboprophylaxis, but at a cost of a two-fold increase in major bleeding (MB) [12]. Antithrombotic guidelines for the overall population of medically ill patients have recommended against routine use of extended thromboprophylaxis with DOACs without reference to specific subgroups that may benefit from such a strategy, with the exception of one guideline (International Union of Angiology) that recommended an individualized approach for extended thromboprophylaxis [13,14,15].

There have been recent developments and new data in the field of extended thromboprophylaxis in medically ill patients that have led to an improved safety profile of DOACs and the establishment of net clinical benefit in key patient subgroups. These developments led to recent FDA approvals of both betrixaban and rivaroxaban for both in-hospital and extended thromboprophylaxis in key subgroups of medically ill patients [16,17]. These approvals have potential in preventing both VTE-related morbidity and mortality and associated risks of arterial thromboembolism in this population [18,19,20]. This article aims to review the most recent concepts of predicting and preventing VTE and to discuss emerging paradigms of extended thromboprophylaxis in key patient subgroups of hospitalized medically ill patients utilizing an individualized, risk-adapted approach.

## 2. Extended Thromboprophylaxis during the Post-Hospital Discharge Period

While most VTE-prevention efforts in hospitalized medically-ill patients have been directed towards the period of acute hospitalization, studies now show that at least 60% of all VTE events occur in the post-hospital discharge period [8,21]. During the first 21-days following discharge, the rate of symptomatic VTE more than doubles and there is an associated 5-fold increase in fatal pulmonary embolism (PE) within 45 days [21]. Despite this being a high-risk period with significant health consequences, the benefits of extended duration thromboprophylaxis remained uncertain among hospital-based health care providers, as reflected in antithrombotic guidelines [13,15]. There have been five studies on extended thromboprophylaxis in medically ill patients four studies comparing extended prophylaxis with enoxaparin 40 mg daily, apixaban 2.5 mg twice daily, rivaroxaban 10 mg daily, and betrixaban 80 mg daily, to standard of care enoxaparin 40 mg daily given for 6 to 14 days that used screening ultrasonography of the lower limbs to assess deep venous thrombosis (DVT) [9,10,11,18,19], and one study comparing extended thromboprophylaxis with rivaroxaban post hospital discharge with placebo that used symptomatic VTE and VTE-related mortality as the primary efficacy endpoint [19]. The results of these studies were mixed.

The Extended Prophylaxis for Venous Thromboembolism in Acutely Ill Medical Patients with Prolonged Immobilization trial (EXCLAIM trial) revealed a net clinical benefit with the use of extended-duration enoxaparin in patients with severe immobility, age older than 75 years, and female sex, but only after a major protocol amendment during the study which rendered the results difficult to interpret [9]. In the Apixaban versus Enoxaparin for Thromboprophylaxis in Medically Ill Patients trial (ADOPT trial), an overall low VTE risk population was identified, and apixaban failed to show efficacy and was associated with significantly more MB events [10]. The Rivaroxaban for Thromboprophylaxis in Acutely Ill Medical Patients trial (MAGELLAN trial) identified a higher VTE risk population and saw a significant reduction in VTE risk with extended-duration rivaroxaban compared to enoxaparin (Relative Risk (RR) 0.77; 95% CI 0.62–0.96) but was associated with an almost 3-fold increase in the risk of MB, including a numerically greater number of fatal bleeds [11]. The Extended Thromboprophylaxis with Betrixaban in Acutely Ill Medical Patients trial (APEX trial) with the DOAC betrixaban also identified a high VTE risk population and narrowly failed to show efficacy in the primary efficacy outcome utilizing a defined patient subgroup, but a pre-specified exploratory analysis provided evidence suggesting a benefit for betrixaban in the total population (RR 0.76; 95% CI 0.63–0.97) [18]. Importantly there was no increase in the risk of MB (0.57% vs. 0.67%, *p* = 0.55), although there was an increase in major or clinically-relevant non-major bleeding (3.1% vs. 1.6%, *p* < 0.001) [18]. A bivariate analysis of this trial revealed a net clinical benefit of −0.51% (95% CI −0.89% to −0.1%) over enoxaparin [22]. The Rivaroxaban for Thromboprophylaxis after Hospitalization for Medical Illness trial (MARINER trial) failed to meet its primary efficacy endpoint but revealed significant reductions in the pre-specified secondary outcomes of symptomatic non-fatal VTE (Hazards Ratio (HR) 0.44; 95% CI 0.22–0.89) and symptomatic VTE and all-cause mortality (HR 0.73, 95% CI 0.54–0.97, *p* = 0.033) [19]. The incidence of MB in both groups was very low (0.28% vs. 0.15, HR 1.88; 95% CI 0.84–4.23), although there was an increase in clinically relevant non-major bleeding (1.42% vs. 0.85%, HR 1.66; 95% CI 1.17–2.35) [19]. Furthermore, the primary efficacy endpoint was met in a sub-group of patients given a 3–6-day course of rivaroxaban (HR 0.55; 95% CI 0.31–0.97), which reflects current LOS in USA hospitals [6,19]. Importantly, a post-hoc analysis of the MAGELLAN trial that excluded approximately 20% of the study population by removing the five key bleeding risk factors used to identify a low bleed risk population in the MARINER trial (bronchiectasis/pulmonary cavitation, active cancer, active gastroduodenal ulcer/history of bleeding within 3 months, and dual antiplatelet therapy) maintained the efficacy of rivaroxaban but reduced the major bleed rates by half so that MB was not significantly worse with rivaroxaban (Day 10, RR 1.19, 95% CI 0.54–2.65; Day 35 RR 1.48, 95% CI 0.77–2.84), in addition to dramatically reducing the rates of fatal bleeding in both treatment phases [20].

Based on a favorable net clinical benefit found in the modified intent-to-treat population in the APEX trial of which there was an evaluable efficacy outcome event, as well as a favorable net clinical benefit in the MAGELLAN subpopulation in which the high bleed risk subgroup with five key bleed risk factors was removed, the FDA has approved both betrixaban 160 mg per os (PO) followed by 80 mg PO daily and more recently rivaroxaban 10 mg PO daily for both inpatient as well as extended post-hospital discharge thromboprophylaxis (31 days up to 39 days) in hospitalized medically ill patients [16,17]. Patients included in these trials were ≥40 years with an acute medical illness, immobility, and had added VTE risk factors.

## 3. Predicting High VTE Risk Medically Ill Patients and Use of Risk Assessment Models

Recent studies have identified key VTE risk factors that place medically ill patients at high risk for VTE post-hospital discharge, and therefore, are more likely to benefit from extended thromboprophylaxis, as shown in Table 1. These key VTE risk factors have either been incorporated individually, as seen in the MAGELLAN and APEX trials, or used as part of a scored and weighted VTE risk assessment model (RAM), such as the modified International Medical Prevention Registry on Venous Thromboembolism (IMPROVE) VTE RAM, as used in the MARINER trial (Table 2). This validated, evidence-derived RAM is a tool that can help guide physicians in the decision-making process of assessing an individual’s VTE risk. The MAGELLAN trial included added VTE risk factors such as age ≥ 75 years, severe varicosities, chronic venous insufficiency, history of malignancy, history of DVT/PE, history of heart failure, thrombophilia, recent major surgery or serious trauma, the use of hormone replacement therapy, BMI ≥ 35 kg/m^2^, and acute infectious disease contributing to hospitalization [11]. A post-hoc analysis also identified an elevated D-dimer (Dd) > 2 times the upper limit of normal as an additive VTE risk factor [23]. The APEX trial included age ≥ 75 years, a history of cancer or VTE, and an elevated Dd as key additive VTE risk factors [18]. A post-hoc analysis of the APEX trial using a modified IMPROVE VTE RAM that incorporated a score of 2 for elevated Dd (the IMPROVEDD VTE RAM) also identified a population with a >2 fold higher VTE risk (HR 2.74, 95% CI 1.52–4.90) than those with a score of 0–1 [24]. The MARINER trial used a modified IMPROVE VTE score of ≥4 or a score of 2 or 3 with elevated Dd as part of the inclusionary criteria, although the observed VTE incidence in the placebo group (1.1%) was lower than the expected incidence of 2.0%–2.5% [19]. Lastly, a recent sub-analysis of the MAGELLAN database also established that the modified IMPROVE VTE RAM with a cut-off score of ≥4 or a score of 2 or 3 with elevated Dd (as used in the MARINER trial) identified a nearly 3-fold higher VTE risk subpopulation of patients with a significant benefit for extended thromboprophylaxis [25]. The totality of evidence from clinical trials, systematic reviews, and a recent meta-analysis on the subject suggests that key VTE risk factors such as previous history of VTE, cancer, known thrombophilia, elevated Dd, advanced age, critical illness, infections, and severe immobility or lower extremity paresis, either as independent risk factors or as part of a VTE RAM, such as IMPROVE, predict a high VTE risk population that would benefit from extended thromboprophylaxis [22,26]. With respect to severe immobility, there have been various definitions that have focused either on the nature or duration of immobility, including the inability to sustain autonomous walking or greater than 10 m and total bedrest with and without bathroom privileges, although more recent definitions have tied severe immobility to the disease state and initial hospitalization period [27,28].

## 4. The Use of Health Informatics Technology and VTE Risk Assessment

Recent efforts have been made to increase the rate of appropriate thromboprophylaxis in medically ill patients by integrating VTE RAMs into each hospital’s electronic medical record (EMR). Informatics technologies such as “Substitutable Medical Applications, Reusable Technologies SMART on Fast Healthcare Interoperability Resource (FHIR) [29],” now have the potential to tie VTE RAMs to an electronic order entry within the workflow of any EMR and alert physician of a patient’s individualized VTE risk. Randomized trials have shown that the use of these electronic alerts has more than doubled the prescription of thromboprophylaxis compared to standard medical care [30,31,32]. This has been shown to be true during both hospital admission for in-hospital thromboprophylaxis and at hospital discharge for extended thromboprophylaxis [30,31,32]. In addition to doubling the rate of pharmacologic prophylaxis with computer alerts (23.6% versus 13%, *p* < 0.001), one study revealed a significant 41% reduction in the incidence of VTE at 90 days (HR 0.59, 95% CI 0.43–0.81, *p* = 0.001) [30]. Another study revealed a significant increase in the rate of thromboprophylaxis with the use of discharge alerts (22% versus 9.7%, *p* < 0.0001); however, there was no difference in the rate of symptomatic VTE at 90 days (4.5% versus 4.0%, HR 1.12, 95% CI 0.74–1.69) [32]. This study did not mandate a specific recommendation for post-hospital discharge thromboprophylaxis tied to a discharge alert, highlighting the fact that computer alerts need to be associated with an actionable outcome such as order entry for them to be effective. While it has been shown that health informatics technology has potential to alter provider behaviors, further study as to whether this technology alters clinical outcomes is warranted.

## 5. Controversies in Antithrombotic Guidelines for Medically Ill Patients

**Figure 1:**
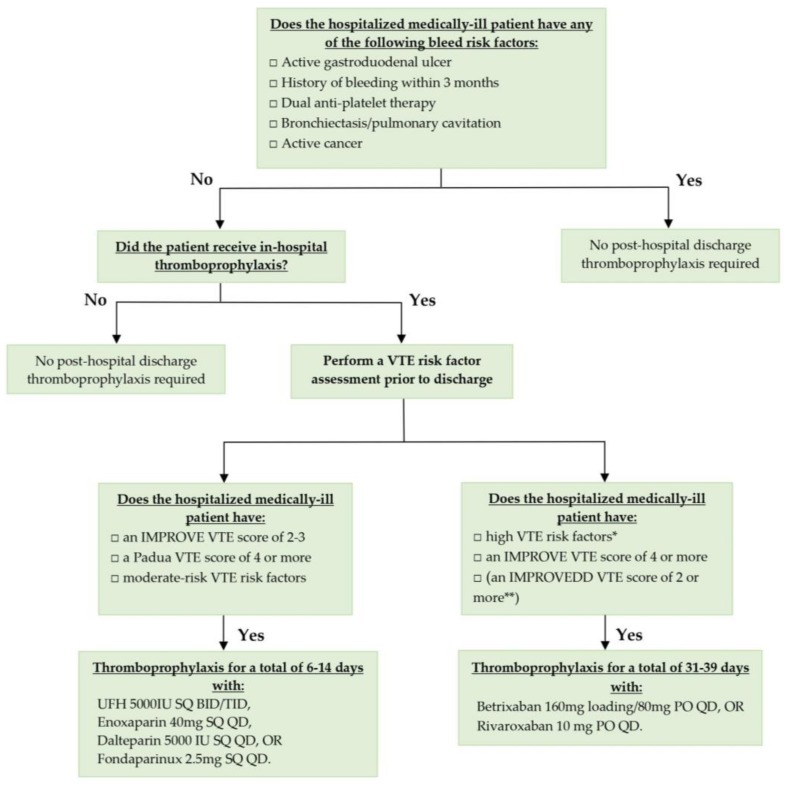
VTE risk assessment and thromboprophylactic strategies for hospitalized medically ill patients prior to discharge. * High VTE risk factors: history of VTE, advanced age, cancer (either active cancer excluding non-melanoma skin cancer or history of cancer within 5 years), severe immobility, stroke with paresis, known thrombophilia, elevated Dd. ** Requires external validation. IMPROVE, International Medical Prevention Registry on Venous Thromboembolism; VTE, venous thromboembolism; UFH, unfractionated heparin; IU, international units; SQ, subcutaneously; BID, twice a day; TID, three times a day; QD, once a day; PO, per os.

The 2018 American Society of Hematology Guidelines provide a strong recommendation for the use of thromboprophylaxis with LMWH only during the period of acute hospitalization, rather than with a DOAC during both acute hospitalization and post-hospital discharge [15]. Furthermore, while forming the basis of these guidelines, the original study endpoint of total VTE was not utilized, which included asymptomatic proximal DVT [15]. It has been consistently shown that a significant association exists between asymptomatic proximal DVT found on screening ultrasonography and an increased risk of all-cause mortality, an association that does not exist with other endpoints such as asymptomatic distal DVT or clinically relevant non-major bleeding [33,34,35]. Therefore, by not including this key primary efficacy endpoint used in the original clinical trials, the guidelines failed to capture the true risk of total VTE and potentially expose high-VTE risk patients to the risks and consequences of VTE in the absence of thromboprophylaxis. These guidelines also failed to establish whether there was net clinical benefit in favor of extended thromboprophylaxis in low bleed risk patient subgroups, as seen in the MAGELLAN subgroup, APEX, and MARINER trials. A more appropriate interpretation of the data—in light of a clear establishment of net clinical benefit and subsequent regulatory approval of both betrixaban and rivaroxaban for extended thromboprophylaxis in key patient subgroups at high VTE and low bleed risk, would be to recommend an individualized, risk-adapted approach to predict which medically-ill patients would benefit the most from post-discharge extended thromboprophylaxis and recommend treatment accordingly. We have included such an evidence-derived algorithm in Figure 1.

## 6. Arterial Thromboembolism and Extended Thromboprophylaxis in the Medically Ill

While an association between VTE and atherosclerosis has been known to exist for some time [36], less is known about the use of extended thromboprophylaxis for the prevention of arterial events, such as myocardial infarction, cardiopulmonary death, and ischemic stroke. Post-hoc analyses of the APEX study revealed that extended thromboprophylaxis with betrixaban significantly reduced all-cause stroke (0.54% versus 0.97%; RR 0.56; 95% CI 0.32–0.96; number needed to treat (NNT) 233) and ischemic stroke (0.48% versus 0.91%; RR 0.53; 95% CI 0.30–0.94; NNT 233) through 77 days of follow-up [37], in addition to significantly reducing irreversible and fatal thromboembolic events (cardiopulmonary death, myocardial infarction, pulmonary embolism, and ischemic stroke) in all patients at 35 to 45 days (4.08% versus 2.90%; HR 0.71; *p* = 0.006; NNT 86), and 77 days (5.17% versus 3.64%; HR 0.70; *p* = 0.002; NNT 65) compared to standard duration thromboprophylaxis [38]. A pre-specified analysis of the MARINER study revealed extended thromboprophylaxis, with the 10 mg dose of rivaroxaban having significantly reduced major and fatal thromboembolic events (including symptomatic VTE, myocardial infarction, ischemic stroke, and cardiovascular death) at 45 days (1.28% versus 1.77%, HR 0.72, 95% CI 0.52–1.00, *p* = 0.049, NNT 204) without an increased risk of MB [39]. Finally, pooled analysis of the MAGELLAN and MARINER trials for extended thromboprophylaxis with rivaroxaban revealed a significant reduction (2.31% versus 1.70%, HR 0.78, NNT 197) in major thromboembolic events and all-cause mortality without an increased risk of critical site/fatal bleeding [40]. The ability for extended duration thromboprophylaxis to prevent irreversible and fatal arterial thromboembolic events is a new area of research that requires further investigation.

## 7. Populational Implications and Future Outlook for Extended Thromboprophylaxis in the Medically Ill

Despite FDA approvals of two DOACs and major populational health consequences of withholding extended thromboprophylaxis in appropriate patients, extended thromboprophylaxis remains an evolving field among regulatory bodies, as The European Medicines Agency (EMA) has rejected betrixaban for this purpose and rivaroxaban is currently under review [41]. Extended thromboprophylaxis in medically ill patients represents a new paradigm for health care professionals as well. Evidence suggests that approximately 25% (and up to 40%) of the 7.2 million medically ill patients in the USA would benefit from extended thromboprophylaxis [42,43]. Applying reductions in symptomatic VTE seen in the APEX trial with betrixaban would result in ~20,000 fewer symptomatic VTEs and the potential to result in 12,000 fewer VTE-related deaths [44]. Data based on the MAGELLAN subgroup suggests that in the EU and USA health systems, a strategy of extended thromboprophylaxis with rivaroxaban has the potential to prevent symptomatic VTE and VTE-related death in approximately 24,000 patients annually at the cost of one half to one fourth that number (approximately 6000 to 12,000 patients) in MB or fatal bleeding events [20]. The advent of oral options for thromboprophylaxis of medically ill patients for both the in-hospital and extended post-hospital discharge period, represents a major advancement in the field of thrombosis, and has the potential to improve patient adherence and patient outcomes [45]. Health informatics technologies are now able to tie VTE RAMs to an electronic order entry within the workflow of any EMR and have potential to reduce the morbidity and mortality associated with hospital-acquired VTE, both in-hospital and in the post-hospital discharge period. The field of extended thromboprophylaxis in the large population of medically ill patients is rapidly evolving, and there is an urgent need to update antithrombotic guidelines based on the best available evidence in order to improve adverse outcomes associated with VTE and minimize harm from chemoprophylactic strategies in appropriately defined patient groups.

## Figures and Tables

**Table 1:** Established individual or model-based venous thromboembolism (VTE) risk factors in hospitalized medically ill patients for extended thromboprophylaxis (factors in bold have more consistently shown high VTE risk in the post-discharge period).

Individual High VTE Risk Factors	Points for the Risk Factor
**History of VTE**	Padua—3 points; IMPROVE—3 points
**Known thrombophilia**	Padua—3 points; IMPROVE—2 points *
Family history of VTE	
**Acute infection**	Padua—1 point
**Malignancy**	Padua—3 points; IMPROVE—2 points **
**Advanced Age**	Padua—1 point; IMPROVE—1 point ***
Severe varicosity/venous insufficiency	
Use of hormone replacement therapy	Padua—1 point
Recent trauma or surgery	Padua—1 point
Morbid obesity	Padua—1 point ****
Congestive heart failure	Padua—1 point
**Stroke with or without Paresis**	Padua—1 point; IMPROVE—2 points *****
**Immobility or Reduced Mobility**	Padua—3 points; IMPROVE—1 point ******
Pregnancy/postpartum	
Acute/chronic lung disease or respiratory failure	Padua—1 point
Acute inflamatory disease or rheumatologic disorder	Padua—1 point
**Shock/ICU/CCU stay**	IMPROVE—1 point
**Elevated Dd (>2 × ULN)**	IMPROVEDD—2 points

VTE Risk Assessment Models (RAMs) for high VTE risk: Padua VTE risk score of 4 or more; IMPROVE VTE risk score of 4 or more or score of 2 or 3 with elevated Dd; IMPROVEDD VTE risk score of 2 or more *******. * A congenital or acquired condition leading to excess risk of thrombosis (e.g., factor V Leiden, lupus anticoagulant, factor C or factor S deficiency). ** May include active cancer (excluding non-melanoma skin cancer) or a history of cancer within 5 years. *** Padua score age > 70 years, IMPROVE score age > 60 years. **** Padua score body mass index (BMI) > 30. ***** IMPROVE score with lower limb paralysis (leg falls to bed by 5 s, but has some effort against gravity, taken from NIH stroke scale). ****** The IMPROVE score’s strict definition is complete immobilization confined to bed or chair ≥ 7 days; the modified definition is complete immobilization ≥ 1 day. ******* The IMPROVEDD VTE score has not undergone external validation. VTE, venous thromboembolism; ICU, intensive care unit; CCU, cardiac care unit; Dd, d-dimer; ULN, upper limit of normal; IMPROVE, International Medical Prevention Registry on Venous Thromboembolism.

**Table 2:** The IMPROVE VTE RAM *.

VTE Risk Factor	Points for the Risk Score
Previous VTE	3
Thrombophilia **	2
Current lower limb paralysis or paresis ***	2
Cancer ****	2
Immobilization *****	1
ICU/CCU stay	1
Age > 60 years	1

Abbreviations: IMPROVE, International Medical Prevention Registry on Venous Thromboembolism; VTE, venous thromboembolism; RAM, risk assessment model; ICU, intensive care unit; CCU, coronary care unit. * A score of 0–1 constitutes low VTE risk; a score of 2–3 constitutes moderate VTE risk; a score of 4 or more constitutes high VTE risk. ** A congenital or acquired condition leading to an excess risk of thrombosis. *** Leg falls to bed by 5 s, but has some effort against gravity (from NIH stroke scale). **** May include active cancer (excluding non-melanoma skin cancer) or a history of cancer within 5 years. ***** Strict definition is complete immobilization confined to bed or chair ≥7 days; modified definition is complete immobilization with or without bathroom privileges ≥1 day.

## References

[1] Anderson F.A., Zayaruzny M., Heit J.A., Fidan D., Cohen A.T. (2007). Estimated annual numbers of US acute-care hospital patients at risk for venous thromboembolism. Am. J. Hematol..

[2] Cohen A.T., Agnelli G., Anderson F.A., Arcelus J.I., Bergqvist D., Brecht J.G., Greer I.A., Heit J.A., Hutchinson J.L., Kakkar A.K. (2007). Venous thromboembolism (VTE) in Europe. The number of VTE events and associated morbidity and mortality. Thromb. Haemost..

[3] Belch J.J., Lowe G.D., Ward A.G., Forbes C.D., Prentice C.R. (1981). Prevention of deep vein thrombosis in medical patients by low-Dose heparin. Scott. Med. J..

[4] Samama M.M., Cohen A.T., Darmon J.Y., Desjardins L., Eldor A., Janbon C., Leizorovicz A., Nguyen H., Olsson C.G., Turpie A.G. (1999). A comparison of enoxaparin with placebo for the prevention of venous thromboembolism in acutely ill medical patients. Prophylaxis in Medical Patients with Enoxaparin Study Group. N. Engl. J. Med..

[5] Cohen A.T., Davidson B.L., Gallus A.S., Lassen M.R., Prins M.H., Tomkowski W., Turpie A.G., Egberts J.F., Lensing A.W., Investigators A. (2006). Efficacy and safety of fondaparinux for the prevention of venous thromboembolism in older acute medical patients: Randomised placebo controlled trial. BMJ.

[6] Flanders S.A., Greene M.T., Grant P., Kaatz S., Paje D., Lee B., Barron J., Chopra V., Share D., Bernstein S.J. (2014). Hospital performance for pharmacologic venous thromboembolism prophylaxis and rate of venous thromboembolism: A cohort study. JAMA Intern. Med..

[7] Mahan C.E., Fisher M.D., Mills R.M., Fields L.E., Stephenson J.J., Fu A.C., Spyropoulos A.C. (2013). Thromboprophylaxis patterns, risk factors, and outcomes of care in the medically ill patient population. Thromb. Res..

[8] Amin A.N., Varker H., Princic N., Lin J., Thompson S., Johnston S. (2012). Duration of venous thromboembolism risk across a continuum in medically ill hospitalized patients. J. Hosp. Med..

[9] Hull R.D., Schellong S.M., Tapson V.F., Monreal M., Samama M.M., Nicol P., Vicaut E., Turpie A.G., Yusen R.D., Study E.E.P. (2010). Extended-Duration venous thromboembolism prophylaxis in acutely ill medical patients with recently reduced mobility: A randomized trial. Ann. Intern. Med..

[10] Goldhaber S.Z., Leizorovicz A., Kakkar A.K., Haas S.K., Merli G., Knabb R.M., Weitz J.I., Investigators A.T. (2011). Apixaban versus enoxaparin for thromboprophylaxis in medically ill patients. N. Engl. J. Med..

[11] Cohen A.T., Spiro T.E., Büller H.R., Haskell L., Hu D., Hull R., Mebazaa A., Merli G., Schellong S., Spyropoulos A.C. (2013). Rivaroxaban for thromboprophylaxis in acutely ill medical patients. N. Engl. J. Med..

[12] Bajaj N.S., Vaduganathan M., Qamar A., Gupta K., Gupta A., Golwala H., Butler J., Goldhaber S.Z., Mehra M.R. (2019). Extended prophylaxis for venous thromboembolism after hospitalization for medical illness: A trial sequential and cumulative meta-Analysis. PLoS Med..

[13] Kahn S.R., Lim W., Dunn A.S., Cushman M., Dentali F., Akl E.A., Cook D.J., Balekian A.A., Klein R.C., Le H. (2012). Prevention of VTE in nonsurgical patients: Antithrombotic Therapy and Prevention of Thrombosis, 9th ed: American College of Chest Physicians Evidence-Based Clinical Practice Guidelines. Chest.

[14] Nicolaides A.N., Fareed J., Kakkar A.K., Comerota A.J., Goldhaber S.Z., Hull R., Myers K., Samama M., Fletcher J., Kalodiki E. (2013). Prevention and treatment of venous thromboembolism—International Consensus Statement. Int. Angiol..

[15] Schünemann H.J., Cushman M., Burnett A.E., Kahn S.R., Beyer-Westendorf J., Spencer F.A., Rezende S.M., Zakai N.A., Bauer K.A., Dentali F. (2018). American Society of Hematology 2018 guidelines for management of venous thromboembolism: Prophylaxis for hospitalized and nonhospitalized medical patients. Blood Adv..

[16] The U.S. Food and Drug Administration FDA Approved Betrixaban (BEVYXXA, Portola) for the Prophylaxis of Venous Thromboembolism (VTE) in Adult Patients. https://www.fda.gov/drugs/resources-information-approved-drugs/fda-approved-betrixaban-bevyxxa-portola-prophylaxis-venous-thromboembolism-vte-adult-patients.

[17] Johnson J.P.C. FDA Approves XARELTO^®^ (rivaroxaban) to Help Prevent Blood Clots in Acutely Ill Medical Patients. https://www.cathlabdigest.com/content/us-fda-approves-xarelto-rivaroxaban-help-prevent-blood-clots-acutely-ill-medical-patients.

[18] Cohen A.T., Harrington R.A., Goldhaber S.Z., Hull R.D., Wiens B.L., Gold A., Hernandez A.F., Gibson C.M., Investigators A. (2016). Extended Thromboprophylaxis with Betrixaban in Acutely Ill Medical Patients. N. Engl. J. Med..

[19] Spyropoulos A.C., Ageno W., Albers G.W., Elliott C.G., Halperin J.L., Hiatt W.R., Maynard G.A., Steg P.G., Weitz J.I., Suh E. (2018). Rivaroxaban for Thromboprophylaxis after Hospitalization for Medical Illness. N. Engl. J. Med..

[20] Spyropoulos A.C., Lipardi C., Xu J., Lu W., Suh E., Yuan Z., Levitan B., Sugarmann C., De Sanctis Y., Spiro T.E. (2019). Improved Benefit Risk Profile of Rivaroxaban in a Subpopulation of the MAGELLAN Study. Clin. Appl. Thromb. Hemost..

[21] Spyropoulos A.C., Anderson F.A., FitzGerald G., Decousus H., Pini M., Chong B.H., Zotz R.B., Bergmann J.F., Tapson V., Froehlich J.B. (2011). Predictive and associative models to identify hospitalized medical patients at risk for VTE. Chest.

[22] Chi G., Goldhaber S.Z., Kittelson J.M., Turpie A.G.G., Hernandez A.F., Hull R.D., Gold A., Curnutte J.T., Cohen A.T., Harrington R.A. (2017). Effect of extended-duration thromboprophylaxis on venous thromboembolism and major bleeding among acutely ill hospitalized medical patients: A bivariate analysis. J. Thromb. Haemost..

[23] Cohen A.T., Spiro T.E., Spyropoulos A.C., Desanctis Y.H., Homering M., Büller H.R., Haskell L., Hu D., Hull R., Mebazaa A. (2014). D-dimer as a predictor of venous thromboembolism in acutely ill, hospitalized patients: A subanalysis of the randomized controlled MAGELLAN trial. J. Thromb. Haemost..

[24] Gibson C.M., Spyropoulos A.C., Cohen A.T., Hull R.D., Goldhaber S.Z., Yusen R.D., Hernandez A.F., Korjian S., Daaboul Y., Gold A. (2017). The IMPROVEDD VTE Risk Score: Incorporation of D-Dimer into the IMPROVE Score to Improve Venous Thromboembolism Risk Stratification. TH Open.

[25] Spyropoulos A.C., Lipardi C., Xu J., Peluso C., Spiro T.E., De Sanctis Y., Barnathan E.S., Raskob G.E. (2020). Modified IMPROVE VTE Risk Score and Elevated D-Dimer Identify a High Venous Thromboembolism Risk in Acutely Ill Medical Population for Extended Thromboprophylaxis. TH Open.

[26] Darzi A.J., Karam S.G., Charide R., Etxeandia Ikobaltzeta I., Cushman M., Gould M.K., Mbuagbaw L., Spencer F., Spyropoulos A., Streiff M.B. (2020). Prognostic factors for VTE and Bleeding in Hospitalized Medical Patients: A systematic review and meta-analysis. Blood.

[27] Hull R.D. (2013). Relevance of immobility and importance of risk assessment management for medically ill patients. Clin. Appl. Thromb. Hemost..

[28] Raskob G.E., Spyropoulos A.C., Zrubek J., Ageno W., Albers G., Elliott C.G., Halperin J., Haskell L., Hiatt W.R., Maynard G.A. (2016). The MARINER trial of rivaroxaban after hospital discharge for medical patients at high risk of VTE. Design, rationale, and clinical implications. Thromb. Haemost..

[29] Fhir H. SMART Application Launch Framework Implementation Guide Release 1.0.0. http://www.hl7.org/fhir/smart-app-launch/.

[30] Kucher N., Koo S., Quiroz R., Cooper J.M., Paterno M.D., Soukonnikov B., Goldhaber S.Z. (2005). Electronic alerts to prevent venous thromboembolism among hospitalized patients. N. Engl. J. Med..

[31] Piazza G., Goldhaber S.Z. (2009). Computerized decision support for the cardiovascular clinician: Applications for venous thromboembolism prevention and beyond. Circulation.

[32] Piazza G., Anderson F.A., Ortel T.L., Cox M.J., Rosenberg D.J., Rahimian S., Pendergast W.J., McLaren G.D., Welker J.A., Akus J.J. (2013). Randomized trial of physician alerts for thromboprophylaxis after discharge. Am. J. Med..

[33] Vaitkus P.T., Leizorovicz A., Cohen A.T., Turpie A.G., Olsson C.G., Goldhaber S.Z., PREVENT Medical Thromboprophylaxis Study Group (2005). Mortality rates and risk factors for asymptomatic deep vein thrombosis in medical patients. Thromb. Haemost..

[34] Kalayci A., Gibson C.M., Chi G., Yee M.K., Korjian S., Datta S., Nafee T., Gurin M., Haroian N., Qamar I. (2018). Asymptomatic Deep Vein Thrombosis is Associated with an Increased Risk of Death: Insights from the APEX Trial. Thromb. Haemost..

[35] Raskob G., Spyropoulos A., Cohen A., Weitz J., Ageno W., De Sanctis Y., Lu W., Xu J., Albanese J., Sugarmann C. (2019). Increased Risk of Death in Acutely ill Medical Patients with Asymptomatic Proximal Deep Vein Thrombosis or Symptomatic Venous Thromboembolism: Insights from the Magellan Study. Blood.

[36] Prandoni P., Bilora F., Marchiori A., Bernardi E., Petrobelli F., Lensing A.W., Prins M.H., Girolami A. (2003). An association between atherosclerosis and venous thrombosis. N. Engl. J. Med..

[37] Gibson C.M., Chi G., Halaby R., Korjian S., Daaboul Y., Jain P., Arbetter D., Goldhaber S.Z., Hull R., Hernandez A.F. (2017). Extended-Duration Betrixaban Reduces the Risk of Stroke Versus Standard-Dose Enoxaparin Among Hospitalized Medically Ill Patients: An APEX Trial Substudy (Acute Medically Ill Venous Thromboembolism Prevention With Extended Duration Betrixaban). Circulation.

[38] Gibson C.M., Korjian S., Chi G., Daaboul Y., Jain P., Arbetter D., Goldhaber S.Z., Hull R., Hernandez A.F., Lopes R.D. (2017). Comparison of Fatal or Irreversible Events With Extended-Duration Betrixaban Versus Standard Dose Enoxaparin in Acutely Ill Medical Patients: An APEX Trial Substudy. J. Am. Heart Assoc..

[39] Spyropoulos A., Ageno W., Albers G., Elliot C., Halperin J., Hiatt W., Maynard G., Steg G., Weitz J., Suh E. (2019). Extended-Duration Thromboprophylaxis with Rivaroxaban Reduces the Risk of Major and Fatal Vascular Events in Hospitalized Medically Ill. Patients.

[40] Raskob G., Spyropoulos A., Cohen A., Spiro T., Lu W., Levitan B., Young S.E., Barnathan E. (2019). Abstract 12863: Rivaroxaban for Extended Thromboprophylaxis After Hospitalization for Medical Illness: Pooled Analysis of Mortality and Major Thromboembolic Events in 16,496 Patients From the MAGELLAN and MARINER Trials. Circulation.

[41] Agency E.M. Dexxience. https://www.ema.europa.eu/en/medicines/human/EPAR/dexxience.

[42] Miao B., Chalupadi B., Clark B., Descoteaux A., Huang D., Ilham S., Ly B., Spyropoulos A.C., Coleman C.I. (2019). Proportion of US Hospitalized Medically Ill Patients Who May Qualify for Extended Thromboprophylaxis. Clin. Appl. Thromb. Hemost..

[43] Martin A.C., Huang W., Goldhaber S.Z., Hull R.D., Hernandez A.F., Gibson C.M., Anderson F.A., Cohen A.T. (2019). Estimation of Acutely Ill Medical Patients at Venous Thromboembolism Risk Eligible for Extended Thromboprophylaxis Using APEX Criteria in US Hospitals. Clin. Appl. Thromb. Hemost..

[44] Cohen A.T. (2018). Extended thromboprophylaxis with betrixaban: A new standard for acute medically ill patients. Eur. Heart J. Suppl..

[45] Owodunni O.P., Lau B.D., Streiff M.B., Kraus P.S., Hobson D.B., Shaffer D.L., Webster K.L.W., Kia M.V., Holzmueller C.G., Haut E.R. (2019). What the 2018 ASH venous thromboembolism guidelines omitted: Nonadministration of pharmacologic prophylaxis in hospitalized patients. Blood Adv..

